# A Maximum Flow-Based Approach to Prioritize Drugs for Drug Repurposing of Chronic Diseases

**DOI:** 10.3390/life11111115

**Published:** 2021-10-20

**Authors:** Md. Mohaiminul Islam, Yang Wang, Pingzhao Hu

**Affiliations:** 1 Department of Computer Science, University of Manitoba, Winnipeg, MB R3T 2N2, Canada; islammm5@myumanitoba.ca (M.M.I.); yang.wang@umanitoba.ca (Y.W.); 2 Department of Biochemistry and Medical Genetics, University of Manitoba, Winnipeg, MB R3T 2N2, Canada; 3 Department of Electrical Computer Engineering, University of Manitoba, Winnipeg, MB R3T 2N2, Canada; 4 CancerCare Manitoba Research Institute, Winnipeg, MB R3T 2N2, Canada

**Keywords:** drug–target interactions, protein–protein interactions, chronic diseases, drug repurposing, maximum flow

## Abstract

The discovery of new drugs is required in the time of global aging and increasing populations. Traditional drug development strategies are expensive, time-consuming, and have high risks. Thus, drug repurposing, which treats new/other diseases using existing drugs, has become a very admired tactic. It can also be referred to as the re-investigation of the existing drugs that failed to indicate the usefulness for the new diseases. Previously published literature used maximum flow approaches to identify new drug targets for drug-resistant infectious diseases but not for drug repurposing. Therefore, we are proposing a maximum flow-based protein–protein interactions (PPIs) network analysis approach to identify new drug targets (proteins) from the targets of the FDA (Food and Drug Administration) drugs and their associated drugs for chronic diseases (such as breast cancer, inflammatory bowel disease (IBD), and chronic obstructive pulmonary disease (COPD)) treatment. Experimental results showed that we have successfully turned the drug repurposing into a maximum flow problem. Our top candidates of drug repurposing, Guanidine, Dasatinib, and Phenethyl Isothiocyanate for breast cancer, IBD, and COPD were experimentally validated by other independent research as the potential candidate drugs for these diseases, respectively. This shows the usefulness of the proposed maximum flow approach for drug repurposing.

## 1. Introduction

Chronic diseases are usually defined as the diseases that are persistent or long-lasting and require ongoing medical attention. There are many different types of chronic diseases. For example, breast cancer starts from the breast cancer cells. However, it can also spread to other parts of the body. Breast cancer is referred to as the most frequently identified cancer in women. This is the second prominent reason for cancer death among women [1]. Of note, cancer is a multistage disease [2], increasing the mortality rate among people worldwide [3]. Several breast cancer treatment techniques are available, such as surgery, chemotherapy, radiation, and hormone therapy. Often a combination of these treatments is used in practice [4]. Other chronic diseases, such as inflammatory bowel disease (IBD) and chronic obstructive pulmonary disease (COPD), are usually consequences of many environmental and genomic factors. IBD is a chronic disease that includes both ulcerative colitis and Crohn’s disease, and it lasts for a very long time. IBD results in a significant burden to our society and families. IBD triggers segments of the bowel to get red and swollen. IBD treatment involves medicines, diet modifications, and occasionally surgery [5]. The goal of such treatment options is to reduce the inflammation associated with IBD. In the long term, existing treatments may achieve reduced risks of IBD complications. COPD is a chronic lung disease that causes breathing problems. COPD is the main reason for respiratory mortality worldwide [6]. Current treatment options include lung transplants, quitting smoking, and inhalers. However, these strategies can only assist in lessening the progression of COPD. The fundamental cause of COPD is smoking [7]. Patients may not know about the disease initially, but the condition worsens over time, such as with severe breathing problems during simple tasks, e.g., walking.

There is a pressing need to identify potential drug targets and their drugs for developing personalized treatments for chronic diseases. However, new drug development takes a very long time and is extremely expensive. Usually, this type of approach takes 10–15 years and $1 billion [8]. Nevertheless, we can save time and money using old drugs for new usages called drug repurposing or repositioning. This is a helpful technique to find different indications for current medications. For example, in 2020, COVID-19 infections from the novel coronavirus became a primary worldwide public health concern [9]. As a result, it was declared as a global pandemic in 2020 [10]. The pandemic created an emergency to develop vaccines or therapeutic treatment for COVID-19 infections. However, there were no available confirmed drugs to treat COVID-19 infections. Therefore, the drug repurposing technique was used to obtain a new drug from the existing FDA-approved drugs [11,12].

There are different types of approaches to identify new indications of an FDA-approved drug, such as network-based [13,14], and machine learning (ML)-based [15,16] approaches.

A biological network consists of a massive number of nodes and interactions among them. A gene can easily make a subnetwork including drug targets, and these drug targets act as the bridge between this subnetwork and the original network. We can identify the risk genes of a given disease and the associated drug targets in a biological network to remove the bridge connection between the subnetwork containing the risk genes and the original network. Therefore, we can potentially treat the disease using drugs associated with the drug targets responsible for the disease’s risk genes in the network.

A network-based approach tries to find a subnetwork that provides an insight into the relationship between drugs and disease genes. For example, Cheng et al. [17] proposed a network-based system to list the drug targets using three different inference algorithms, which are drug resemblance in any network, protein correspondence in any network, and recognized drug target within a bipartite network. 

Yeh et al. [18] first proposed a maximum flow approach to predict a set of drugs as new effective drug targets for the treatment of prostate cancer. The idea is that the candidate proteins for a drug target with a higher flow value to the risk genes have more influence on risk genes than other candidates for the drug targets. They used microarray data [19] and an interactome (PPI) network [20] of prostate cancer to build their prediction model. Next, they used the shortest path algorithm [21] to perform a maximum flow method within their network and successfully identified 20 drug targets to reuse. These drug targets were validated using other available literature that published these same drug targets for prostate cancer.

Melak et al. [22] also used the idea of the maximum flow approach to prioritize a set of drug targets to reduce the expression of tuberculosis disease from a list of known drug targets. Yeh et al. [18] used the Pearson correlation coefficient and gene expression changes between genes to calculate the weight of the edges of their PPI network. However, Melak et al. [22] used a PPI network from STRING which includes the associated weights for the edges. Thus, Yeh et al. [18] and Melak et al. [22] showed that proteins with the maximum flow to the risk genes in the PPI network could be used as targets for developing drugs to treat diseases.

This study aims to apply the maximum flow technique to a PPI network with a set of breast cancer, IBD, and COPD risk genes to identify new breast cancer, IBD, and COPD drugs, respectively, from a list of FDA-approved medications. We hypothesize that identifying new drugs from the existing drugs (i.e., drug repurposing) for breast cancer, IBD, and COPD can be converted into a maximum flow problem using a human interactome network (i.e., a PPI network). Furthermore, it is believed that drug targets X (proteins) connected with risk genes through a higher flow value have more impact on these risk genes than other drug targets. Therefore, these Xs can be used as potential targets for drug development for the disease’s treatment. Furthermore, deletion of these Xs from the PPI network will disrupt the communication among the risk genes and proteins. Therefore, this study aims to identify a set of strongly correlated proteins with the disease risk genes from a PPI network using a maximum flow approach. Later, we can identify new candidate drugs for repurposing to treat breast cancer, IBD, and COPD associated with these targets using a drug-target interaction network.

## 2. Materials and Methods

### 2.1. Datasets

#### 2.1.1. Protein–Protein Interaction (PPI) Network

**Figure 1:**
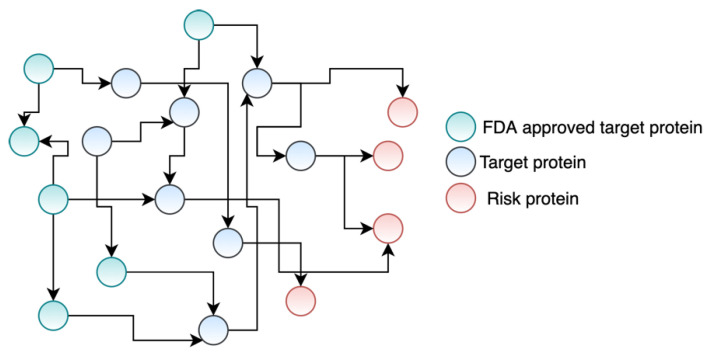
An example of our PPI network. The network shows the interactions among the FDA-approved drug targets (i.e., proteins), potential drug targets, and disease-associated risk proteins/genes.

We collected a comprehensive biological network [23] which includes 140,899 interactions among the 13,365 human proteins (genes). We used this biological network (Figure 1) to conduct our experiments. 

#### 2.1.2. Drug-Target Interactions (DTIs) Network

We extracted 2390 FDA-approved drug targets (DTs) in human from DrugBank [24]. However, the PPIs network described in Section 2.1.1 contains only 1926 DTs among these 2390 FDA-approved DTs. We also collected the DTIs network, which has ~13,000 DTIs among 5049 unique drugs and 3099 unique targets from the DrugBank. 

#### 2.1.3. Risk Genes

In this study, we focused our drug repurposing on the above-mentioned three diseases (breast cancer, IBD, and COPD) since they have a relatively large number of disease-specific risk genes identified from genome-wide association studies (GWAS) as described below. These risk genes make the application of the maximum flow technique to drug repurposing possible in this study. GWAS have already discovered more than 200 breast cancer risk loci. For example, Baxter et al. [25] were able to mark 63 loci and identified 110 known target genes at 33 loci. In addition, Wu et al. [26] identified 179 significant genes associated with breast cancer risk. Thus, we have collected in total 289 breast cancer risk genes from these two studies. 

Previously published genomic studies identified 215 risk loci to explain the fundamental molecular biology of IBD [27]. In addition, Katrina et al. [27] marked three additional loci which have therapeutic targets in IBD. They have also prioritized 811 IBD risk genes from 240 risk variants. 

A GWAS in the United Kingdom by Sakornsakolpat et al. [6] identified 82 loci associated with COPD or function. Among them, 47 loci were already known as risk loci of COPD. Of note, Sakornsakolpat et al. [6] have identified 156 COPD risk genes from these 82 loci. 

Hence, we have collected 289, 811, and 156 risk genes responsible for breast cancer, IBD, and COPD, respectively, from the earlier studies to validate the usefulness of our proposed drug repurposing method.

### 2.2. The Maximum Flow Algorithm for Drug Repurposing

**Figure 2:**
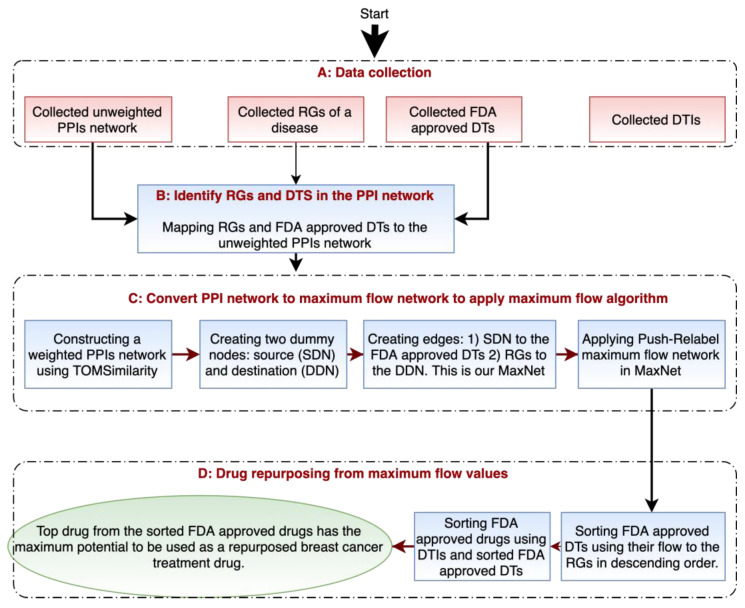
Analysis pipeline for the maximum flow approach to prioritize drugs for drug repurposing (taking breast cancer as an example). (**A**) Shows the types of data we collected for our experiments. (**B**) We mapped each target protein in the PPI network to be either a risk gene, FDA-approved drug target, or potential candidate target. (**C**) Shows the construction of maximum flow network from the collected PPI network to apply the Push-Relable maximum flow algorithm. (**D**) Shows the steps to repurpose existing drugs based on the maximum flow values of each target protein.

The analysis pipeline for drug repurposing includes multiple steps, as shown in Figure 2 (taking breast cancer as an example). Below we explain the steps in more detail.

#### 2.2.1. Constructing the Maximum Flow Network

Mapping drug targets and risk genes to the PPIs network: We first mapped the 1926 FDA-approved DTs (FDA_DT) and risk genes (RGs) of a specific disease to the unweighted PPIs network (refers to a graph where edges do not have weights, and there is only one edge between any two nodes). 

Constructing weighted PPIs network: We used TOMSimilarity (topological overlap matrix similarity) [19] to calculate the weight of edges between genes, and we used Equation (1) to get TOMSimilarity between two nodes in our network.

(1) $$TOMSimilarity\;\left(x,y\right)=\frac{\left|N_{neighbor}\left(x\right)\cap N_{neighbor}\left(y\right)\right|+A_{xy}}{\min\left(|N_{neighbor}\left(x\right)\left|,\;|N_{neighbor}\left(y\right)\right|\right)+1-A_{xy}}$$

$\mathrm{where}\;N_{neighbor}\left(x\right)$ is the neighbors of x,

$N_{neighbor}\left(y\right)$ is the neighbors of y,

$A_{xy}$ is the value of the adjacency matrix (i.e., one if nodes x and y are connected and zero otherwise),

TOMSimilarity (x,y) is the Topological Overlap Matrix Similarity between the nodes x and y.

Drug repurposing as a maximum flow problem: After the mapping of the drug targets and risk genes, we specified the drug repurposing problem into a maximum flow problem, we (1) created a dummy node SDN (i.e., the source of the network) which was connected with all the FDA_DT; (2) created another dummy node DDN (i.e., the destination of the network) which was connected with all the risk genes; (3) assigned a flow capacity (i.e., weight) using Equation (1) for each of the connections in the network. Flows in the maximum flow network follow the below rules: (1) The input flow is equal to the output flow for any node except the source and destination nodes; (2) for any edge (*e*) in the network, 0 ≤ flow(*e*) ≤ Capacity(*e*); (3) total flow out of the source node is equal to total flow into the destination node.

**Figure 3:**
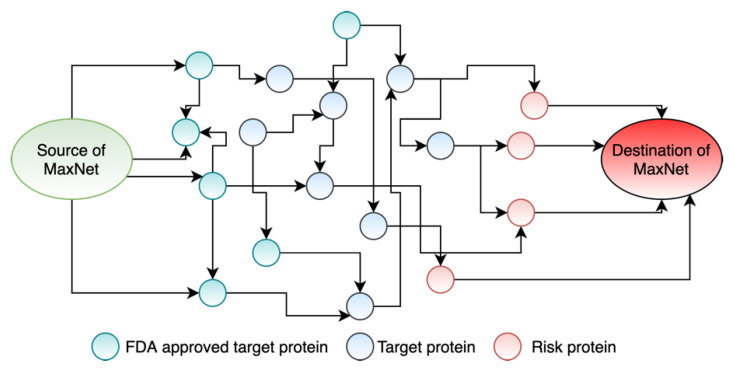
An example of our MaxNet.

However, the connections from the dummy source to the candidate drug targets will have a dummy capacity. Each incoming edge from the dummy source node to a protein (drug target) has a capacity equal to the sum of the capacities of the outgoing edges from that protein (drug targets). Similarly, the connections from the risk genes to the dummy sink node have dummy capacities. Each outgoing edge from a risk gene to the sink node has a capacity equal to the sum of the capacities of the incoming edges to that risk gene. At this point, we had the network named MaxNet (Figure 3) to run the maximum flow algorithm.

#### 2.2.2. Push-Relabel Maximum Flow Algorithm

We used the Push-Relabel maximum flow algorithm [28] in the MaxNet (Figure 3) to maximize the flow amount passed from the FDA-approved drug targets to the risk genes. Algorithm 1 (revised from [29]) shows the Push-Relabel maximum flow algorithm. In addition, this algorithm works with one vertex at a time. Every vertex is associated with two variables: height and excess flow. A vertex can send flows to a lower-height vertex only. The extra flow of a vertex represents the difference between the total in-flow and out-flow of that vertex. Furthermore, each edge is associated with two variables: flow (i.e., current flow through this edge) and capacity (i.e., the maximum flow we can send through this edge). This algorithm sends flows (i.e., PUSH operation) from a node (S) to its adjacent node (D) when the excess flow of D is not equal to zero and the height of D is less than the height of S. If there is no adjacent node of S with lesser height than this algorithm increases the height of S (i.e., RELABEL operation) by the minimum height of the adjacent nodes of S plus 1.
**Algorithm 1** Push-Relabel_MaximumFlow_Algorithm [28].**Input:** PPI, Capacity = C, N = unique nodes of PPI, start_node = SDN, destination_node = DDN. **Output:** Maximum flow between SDN and DDN  (1) FOR i = 1 to length [N]:    a. HeightV [i] = 0//HeightV is height of every vertex    b. FlowV [i] = 0//FlowV is the flow of every vertex  (2) HeightV [start_node] = length [N]   (3) FOR i = 1 to length [PPI]:    a. FlowE [i] = 0//FlowE is the flow of every edge in the PPI  (4) V = adjacentVetex[start_node]  (5) FOR i = 1 to length [V]:    a. FlowV [V[i]] = Capacity [V[i]]    b. excessFlow [V[i]] = Capacity [V[i]]  (6) **PUSH:** FOR i = 1 to length [N]:  If excessFlow [N[i]] ≠ 0: (in the residual graph)     tmpV = adjacentVetex[N[i]]     if HeightV [N[i]] > lowest_height[tmpV]      Push_flow from N[i] to lower height vertices  (7) **RELABEL:** FOR i = 1 to length [N]:  If excessFlow [N[i]] ≠ 0: (in the residual graph)    tmpV = adjacentVetex[N[i]]    if HeightV [N[i]] ≤ lowest_height[tmpV]     HeightV [N[i]] = minimumHeight[tmp]

#### 2.2.3. Drug Repurposing from Maximum Flow Values

After applying the Push-Relabel maximum flow algorithm in our MaxNet network, we sorted all the FDA drug targets into a list L_DTs_ according to their flow value to the risk genes (descending order). Then, we used this sorted list L_DTs_ of the DTs to sort the FDA-approved drugs into a list L_drugs_ using ~13,000 DTIs collected from DrugBank [29]. Hence, according to our hypothesis, the top drugs in L are the most prominent drugs that can be reused to treat the given disease associated with its risk genes.

The whole analysis pipeline of the maximum flow-based drug repurposing is summarized in Algorithm 2.
**Algorithm 2** Pipeline of the maximum flow-based drug repurposing.**Input:** PPI = all the PPIs, FDA_DT = all the FDA approved DTs in PPIs network, DTI = DTIs for FDA_DT, RG = risk genes, W = flow capacity of edges. **Output:** CD = candidate drugs for repurposing for the treatment of breast cancer.  1. FOR i = 1 to length [PPI]:    a. Calculate flow capacity of the edge using Equation (1):      C[i] = TOMSimilarity (PPI[i])   2. CREATE two dummy nodes:    a. source dummy node = SDN and destination dummy node = DDN  3. FOR i = 1 to length [FDA_DT]:    a. Index = length [PPI] + 1    b. CONNECT SDN to FDA_DT[i] and add this interaction in PPI[index]    c. W[index] = sum of the capacities of the outgoing edges from PPI[index]  4. FOR i = 1 to the length of RG:    a. Index = length of PPI + 1    b. CONNECT RG[i] to DDN and add this interaction in PPI[index]    c. C[index] = sum of the capacities of the incoming edges from PPI[index]  5. The nodes in PPIs and their associated outgoing flow value = Push-Relabel_MaximumFlow_Algorithm (PPI, C, SDN, DDN)  6. prioritized_DTs = sort the nodes in PPI in decreasing order of their outgoing flows  7. CD = sort drugs in DTI using prioritized_DTs

## 3. Experimental Results

### 3.1. Mapping Drug Targets and Disease-Specific Risk Genes to the PPIs Network

**Figure 4:**
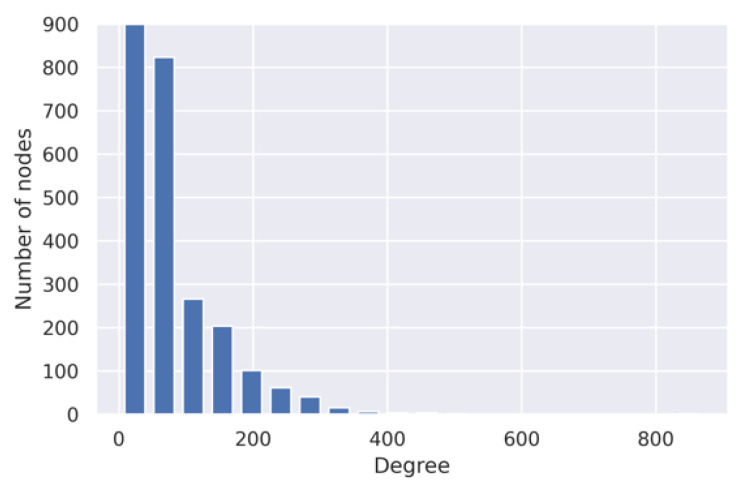
Degree distribution of the PPIs network.

First, we collected the PPI network. This is an unweighted network. So, we calculated topological overlap similarity (TOMSimilarity) to assign weights on the edges. These weights were used as the capacities of the flow through the edges. In this PPI network, we had 1926 FDA-approved DTs. Next, we mapped disease-specific risk genes to this PPI network. The PPIs network contained 155 breast cancer RGs from the 289 breast cancer RGs identified by Baxter et al. [25] and Wu et al. [26]. It also had 565 IBD risk genes among the 811 prioritized IBD risk genes by Katrina et al. [27]. This PPI network also contained 118 COPD risk genes among the 156 COPD risk genes identified by Sakornsakolpa et al. [6]. Table 1 shows several statistical properties of the PPI network. In Table 1, transitivity refers to the probability of adjacent nodes being interconnected. It provides an intuition about the clusters in the network. Of note, in a graph, total triangles represent the total number of triangles formed by any three nodes. In addition, we also showed the PPI network’s degree distribution in Figure 4. Figure 4 indicates that only a few nodes in the PPIs network have a high number of neighbors. This means the PPI network has a small number of hubs.

### 3.2. Weights of the Interactions in PPIs Network

We calculated topological overlap similarity (TOMSimilarity) to assign weights on the edges of the unweighted PPI network. The values of these edge weights ranged from 0 to 1. We used these edge weights as flow capacity for each connection during maximum flow implementation with Algorithm 1.

### 3.3. Formulating Drug Repurposing as a Maximum Flow Network

FDA-approved drug targets are the network sources, while risk genes are the destinations of the network. Hence, we needed to convert this multiple sources and multiple destinations network into a single source and single destination network. To do this, we created a dummy source node and connected this node with 1926 DTs. Similarly, we created a dummy destination node and only connected this sink node with the disease-specific risk genes. As a result, there were no incoming arcs to the source node and no outgoing arcs from the destination node. We calculated the sum of the capacities of the outgoing arcs from a drug target node and put this sum as the capacities on the arcs from the dummy source node to the drug target node. Likewise, we calculated the sum of the capacities of the incoming arcs to a risk gene node and put this sum as the capacities to the arc from the risk gene node to the dummy destination node. We called this network the MaxNet.

### 3.4. Drug Repurposing for Breast Cancer, IBD, and COPD

We created three MaxNets (MaxNet_BC, MaxNet_IBD, and MaxNet_COPD) for breast cancer, IBD, and COPD RGs, respectively. For all three MaxNets, the dummy source node was connected with the 1926 FDA-approved DTS. However, our PPIs network contained only 155 breast cancer RGs, 565 IBD RGs, and 118 COPD RGs. Therefore, we connected the 155 breast cancer RGs with the dummy destination node in the MaxNet_BC, the 565 IBD RGs with the dummy destination node in the MaxNet_IBD, and the 118 COPD RGs with the dummy destination node in the MaxNet_COPD.

We ran the Push-Relabel maximum flow algorithm in all three MaxNets to get the maximum flow values for each node from the dummy source to the dummy destination. First, we extracted three sorted lists of the targets (FDA-approved) based on their outgoing flows from the MaxNet_BC, MaxNet_IBD, and MaxNet_COPD in descending order. Then, we used these sorted lists of targets to sort the drug list using a drug-target interaction network for breast cancer, IBD, and COPD. According to our hypothesis, the top drug in each of these sorted drug lists has the maximum potential to be used as a candidate drug for the treatment of breast cancer, IBD, and COPD, respectively.

### 3.5. Performance Evaluation

We performed a comprehensive literature review to validate our top five repurposed candidates for breast cancer, IBD, and COPD as shown in Table 2, Table 3 and Table 4, respectively.

In addition, we have shown the top 10 prioritized repurposed drugs in the Supplementary Tables S1–S3 for each of these diseases.

### 3.6. Performance Comparison with Other Methods

We used the same datasets to compare the performance of our maximum flow-based drug prioritization with the baseline methods, such as degree, betweenness centrality, closeness centrality, random walk, and page rank (Table 5). Degree centrality refers to the number of incoming links to a node and ranks the risk genes by their degree value. Closeness centrality is defined as the geodesic distance (normalized) for any node to any other node in the network. Finally, the betweenness centrality of a node denotes the number of shortest paths that include this node. First, we used MATLAB functions to calculate degree centrality, closeness centrality, and betweenness centrality from the PPI network for each disease of interest (breast cancer, IBD, and COPD). Then, we sorted each of these lists of targets in descending order. Furthermore, we obtained a sorted list of candidate drugs using these sorted targets and a drug-target interaction dataset. Then we used the python functions of random walk [41] and page rank [42] to calculate the importance of each target associated with breast cancer, IBD, and COPD in the PPI network. Finally, we used sorted random walk [41] and page rank [42] (descending order) lists of targets to identify potential drug repurposing candidates from the drug-target interaction network we collected from the DrugBank database.

## 4. Discussion

Traditional machine learning methods, such as naive Bayesian, support vector machines, and the latest deep neural networks, reveal their effectiveness for drug discovery. Zhao et al. proposed a method that uses drug-induced expression profiles to predict the sign of a disease in psychiatry [43]. Saberian et al. [44] introduced a framework that takes anti-similarity between drugs and a disease as input to train a model. Their model can predict new usage apart from the primary indications of a drug. However, researchers have concerns about using conventional machine learning techniques for this purpose because of the background noisiness and the high-dimensionality nature of the biological data [45]. Hence, Cheng et al. [17] used a chemical structure with the genome sequence to perform the drug and protein resemblance checking. At the same time, they anticipated related drugs might share identical drug targets for a disease. However, they did not find any helpful result from these similarities checking among the drugs. Nevertheless, they concluded that the chemical structure could not be represented as a parameter to identify similar drugs or proteins. Estrada et al. [46] also used a biological network’s global measure such as closeness/betweenness centrality to identify drug targets. They considered a node in the network as the drug target if it has a higher closeness/betweenness centrality value than the other nodes. These measures are based on the shortest paths in the network. In addition, random walk [41] and page rank (the algorithm that Google uses for their search engine) [42] can be used to extract such global measures to identify potential new drug targets. In this study, we adopted a maximum flow-based approach similar to Yeh et al. [18] and Melak et al. [22] to prioritize FDA-approved drugs repurposed for breast cancer, IBD, and COPD.

We used a PPI network [23] to conduct our experiments. The investigators mentioned that these interactions do not contain any interactions estimated from gene expression data. These interactions fall into the following categories: protein–protein interactions (most of the interactions fall into this category), regulatory interactions, protein database, and signaling interactions [47]. However, this PPI network is not weighted. Therefore, we converted our PPI network to a weighted network using TOMSimilarity. We used TOMSimilarity because Langfelder et al. [48] showed its effectiveness as a highly robust measure of network interconnectedness (proximity) for the hierarchical clustering of biological data. TOMSimilarity calculates the topological similarity between two connected proteins (i.e., genes) using an adjacency matrix. Then, we applied the Push-Relabel algorithm to obtain the node importance based on its outflow. This algorithm works locally rather than looking into the entire residual graph (this graph indicates if it is possible to send flows from the source to the destination of the network) to find an augmenting path to send flows.

The primary usage of our most promising candidate drug, “Guanidine” (Table 2), is to treat muscle weakness caused by Eaton-Lambert syndrome. In 2009, Meruling et al. [30] showed that at 0.5 microM, dextran aminoguanidine conjugate killed more than 95% of the breast cancer cells compared to 25% for Adriamycin. The second candidate, “Phenethyl Isothiocyanate” (PEITC) (Table 2), with unique specificity, has promising results for HER2 breast cancer patients. “Caffeine” (Table 2) is primarily used to restore mental alertness when fatigue or drowsiness are present and for the treatment of post-dural lumbar puncture headaches. However, Pantziarka et al. [32] confirmed that caffeine could be used to treat breast cancer. The fourth candidate, “Tamoxifen,” is primarily used for breast cancer. Hence, we showed the top five candidate drugs using our proposed framework in Table 2.

According to our proposed framework, the most promising candidate drug used as the IBD repurposed drug is “Dasatinib” (Table 3). It has been shown that Dasatinib is helpful to decrease the inflammation in a rodent model of colitis [34] for ulcerative colitis type IBD. Therefore, the study concluded that Dasatinib could be a potential candidate for ulcerative colitis treatment. Our second IBD repurposed drug candidate is “Phenethyl Isothiocyanate” (PEITC) (Table 3). PEITC Essential Oil contains more than 95% of PEITC. Therefore, Dey et al. [35] confirmed PEITC essential oil as a potential treatment for ulcerative colitis patients. The third candidate, “Adenosine” (Table 3), is working as a modulator for inflammation (including Crohn’s disease and ulcerative colitis) both in humans and animals [36]. Our last candidate, “Glutamic Acid” (Table 3), was confirmed by [37] as an amino acid is an adjuvant ulcerative colitis type of IBD treatment. Furthermore, the investigators showed that microinjection of this amino acid into the paraventricular nucleus on ulcerative colitis in rats significantly improved anti-oxidation levels. This outcome suggests that glutamic acid is a potential candidate for a therapeutic application of paraventricular nucleus regulation in ulcerative colitis. However, the doses of glutamic acid may change for the naturally-occurring IBD.

The primary usage of Phenethyl Isothiocyanate (PEITC) is the treatment of lung cancer [38]. Nonetheless, our proposed framework considers this drug the most favorable contender in our top five candidate drugs list (Table 4) for COPD. Our next candidate, “Minocycline” (Table 4), is effective as an addition to treatment with cyclophosphamide in reducing the number of lung cancer [39]. The third candidate, “Pseudoephedrine” (Table 4) can also be used for COPD-related diseases such as the treatment of nasal and sinus congestion that is caused by a breathing illness (e.g., bronchitis) [49,50]. Finally, the last candidate, “NADH” (Table 4), improves trial COPD [40], emphasizing a probable helpful treatment for COPD.

From Table 5**,** it is self-evident that our proposed framework outperformed baseline methods (degree centrality, betweenness and closeness centrality, random walk [15], and page rank [42]) in prioritizing drug candidates for disease-specific drug repurposing. A literature review-based validation confirmed that our proposed framework correctly prioritized four out of the top five candidate drugs for drug repurposing for breast cancer, IBD, and COPD, respectively. On the other hand, degree and betweenness centrality methods have only one and two confirmed drug candidates, respectively, to be used as repurposed drugs for COPD only. Closeness centrality has two and one confirmed drug candidates as repurposed drugs for breast cancer and IBD, respectively. The random walk has zero, two, and two confirmed drugs in the predicted top five drugs to treat breast cancer, IBD, and COPD diseases, respectively. However, the page rank approach listed two confirmed drugs for each disease in the top five predicted lists of drugs.

The above literature review-based comparison suggests that our proposed framework can be used for novel drug discovery and drug repurposing. Therefore, it may be promising to use the proposed drug repurposing framework to prioritize candidate disease-specific repurposed drugs and disease-specific primary drugs.

## 5. Conclusions

This study aims to formulate drug repurposing for a specific disease as a maximum flow problem. We used a human interactome network and a set of FDA-approved drug targets along with different disease-specific (breast cancer, IBD, and COPD) risk genes to perform our experiments. We hypothesized that our proposed framework would identify a set of FDA-approved drugs that can be repurposed to treat breast cancer, IBD, and COPD. Experimental results showed that we had identified a prioritized list of drug targets and associated drugs that can be reused to treat these diseases. Furthermore, our proposed framework identified the natural flow to strongly influence the disease genes without any prior knowledge. Finally, we performed a comprehensive literature review to validate our proposed framework’s performance. This validation shows that our proposed framework outperformed other baseline methods regarding the total number of confirmed repurposed drugs. The validation also suggests that our drug repurposing approach can also be used for novel drug discovery.

Future works of this study include experiments and clinical trials with our prioritized lists of candidate drugs. These approaches will confirm whether our candidate drugs have the potential to treat breast cancer, IBD, and COPD, respectively.

## Figures and Tables

**Table 1:** Statistical properties of the PPIs network.

Properties	Values
Number of nodes	13,368
Number of edges	140,899
Transitivity	0.292
Average clustering coefficient	0.173
Edge density	0.002
Average degree	21.08
Total triangles	4,105,272

**Table 2:** The top five repurposed drugs for breast cancer.

Drug Name	Target Protein	Target Gene	Flow Value	Status	Reference
Guanidine	P78352	DLG4	0.0489	Confirmed	[30]
Phenethyl Isothiocyanate	P31946	YWHAB	0.0389	Confirmed	[31]
Caffeine	P78527	PRKDC	0.0363	Confirmed	[32]
Tamoxifen	Q05655	PRKCD	0.0363	Confirmed	[33]
(2S)-2-({6-[(3-Amino-5-chlorophenyl)amino]-9-isopropyl-9H-purin-2-yl}amino)-3-methyl-1-butanol	Q00534	CDK6	0.03319202		

**Table 3:** The top five repurposed drugs for IBD.

Drug Name	Target Protein	Target Gene	Flow Value	Status	Reference
Dasatinib	P12931	SRC	0.08292133	Confirmed	[34]
Phenethyl Isothiocyanate	P31946	YWHAB	0.06112281	Confirmed	[35]
Adenosine-5′	P00558	PGK1	0.04545455	Confirmed	[36]
Acetylsalicylic acid	P54646	PRKAA2	0.03627599		
Glutamic Acid	P07814	EPRS	0.03527291	Confirmed	[37]

**Table 4:** The top five repurposed drugs for COPD.

Drug Name	Target Protein	Target Gene	Flow Value	Status	Reference
Phenethyl Isothiocyanate	P31946	YWHAB	0.05054656	Confirmed	[38]
Minocycline	P42574	CASP3	0.03767546	Confirmed	[39]
Pseudoephedrine	P15336	ATF2	0.03201844	Confirmed	[38]
Methyl 4,6-O-[(1R)-1-carboxyethylidene]-beta-D-galactopyranoside	P02743	APCS	0.03150388		
NADH	O43920	NDUFS5	0.02409639	Confirmed	[40]

**Table 5:** Number of confirmed disease-specific candidates by the baseline approaches for drug repurposing in the list of top five candidate drugs.

Method	Number of Confirmed Candidates in Top 5 Candidate Drug List		
	Breast Cancer	IBD	COPD
Degree centrality	0	0	1
Closeness centrality	2	1	0
Betweenness centrality	0	0	2
Random walk [41]	0	2	2
Page rank [42]	2	2	2
Our proposed framework	4	4	4

## Data Availability

All the datasets used for the experimental analysis are publicly available.

## Supplementary Materials

The following are available online at https://www.mdpi.com/article/10.3390/life11111115/s1, Table S1: The top 10 prioritized repurposed drugs for breast cancer, Table S2: The top 10 prioritized repurposed drugs for IBD, Table S3: The top 10 prioritized repurposed drugs for COPD.
