# Supplementary material for: A Maximum Flow-Based Approach to Prioritize Drugs for Drug Repurposing of Chronic Diseases
Source: Life (Basel). 2021 Oct 20;11(11):1115. doi: 10.3390/life11111115 (PMC8625622; doi:10.3390/life11111115)
Supplement: Supplementary file 1 [file life-11-01115-s001.zip › life-1346560-supplementary.pdf]

Supplementary Information

A maximum flow-based approach to prioritize drugs for drug repurposing of chronic diseases

In this supplementary, we presented the prediction performance of our proposed framework for the top 10 prioritized repurposed drugs for breast cancer (Table S1), Inflammatory bowel disease, i.e., IBD (Table S2) and Chronic Obstructive Pulmonary Disease, i.e., COPD (Table S3).

Table S1: The top 10 prioritized repurposed drugs for breast cancer.

Drug Name	Target protein	Target gene	Flow value	Status	Reference
Guanidine	P78352	DLG4	0.0489	Confirmed	1
Phenethyl Isothiocyanate	P31946	YWHAB	0.0389	Confirmed	2
Caffeine	P78527	PRKDC	0.0363	Confirmed	3
Tamoxifen	Q05655	PRKCD	0.0363	Confirmed	4
(2S)-2-({6-[(3-Amino-5-chlorophenyl)amino]-9-isopropyl-9H-purin-2-yl}amino)-3-methyl-1-butanol	Q00534	CDK6	0.03319202		
Epinephrine	P00441	SOD1	0.03128153		
Purvalanol	P11802	CDK4	0.03128055	Confirmed	5
L-Lysine	Q15046	KARS	0.02918842	Confirmed	6
Phosphoaminophosphonic Acid-Adenylate Ester	P08069	IGF1R	0.02522087	Confirmed	7
Minocycline	P42574	CASP3	0.02489913	Confirmed	8

Table S2. The top 10 prioritized repurposed drugs for the IBD.

Drug Name	Target protein	Target gene	Flow value	Status	Reference
Dasatinib	P12931	SRC	0.08292133	Confirmed	9
Phenethyl Isothiocyanate	P31946	YWHAB	0.06112281	Confirmed	10
Adenosine-5'	P00558	PGK1	0.04545455	Confirmed	11
Acetylsalicylic acid	P54646	PRKAA2	0.03627599		
Glutamic Acid	P07814	EPRS	0.03527291	Confirmed	12
Pyruvic acid	P14618	PKM	0.03305785		
Trimetazidine	P09110	ACAA1	0.03061647	Confirmed	13
ATP	P68400	CSNK2A1	0.03056766	Confirmed	14
Dimethyl fumarate	Q04206	RELA	0.02998375	Confirmed	15
Tamoxifen	P05129	PRKCG	0.02828854		

Table S3: The top 10 prioritized repurposed drugs for COPD.

Drug Name	Target protein	Target gene	Flow value	Status	Reference

Phenethyl Isothiocyanate	P31946	YWHAB	0.05054656	Confirmed	16
Minocycline	P42574	CASP3	0.03767546	Confirmed	17
Pseudoephedrine	P15336	ATF2	0.03201844	Confirmed	16
Methyl 4,6-O-[(1R)-1-carboxyethylidene]-beta-D-galactopyranoside	P02743	APCS	0.03150388		
NADH	O43920	NDUFS5	0.02409639	Confirmed	18
Pyridoxal phosphate	P00505	GOT2	0.02248822	Confirmed	19
Lauric acid	P41235	HNF4A	0.02246764	Confirmed	20
Deferoxamine	P05067	APP	0.02094241	Confirmed	21
Isoprenaline	P27986	PIK3R1	0.02046899	Confirmed	22
Adapalene	P10276	RARA	0.01800372		

It is evident from Table S1, Table S2 and Table S3 that the prediction performance of our proposed framework performance is similar to the performance of the top five prioritized repurposed drugs (Table 2, Table 3 and Table 4 of the main manuscript). In breast cancer, IBD and COPD cases, we have confirmed (validated by literature review) eight, seven and eight drugs, respectively, in the top 10 prioritized repurposed drugs. This performance suggests that further in-vitro validation may confirm the potential clinical usage of our prioritized repurposed drugs.

Reference:

(1) Meurling, L.; Marquez, M.; Nilsson, S.; Holmberg, A. R. Polymer-Conjugated Guanidine Is a Potentially Useful Anti-Tumor Agent. Int. J. Oncol. 2009, 35 (2), 281–285. https://doi.org/10.3892/ijo_00000338.

(2) Gupta, P.; Srivastava, S. K. Antitumor Activity of Phenethyl Isothiocyanate in HER2-Positive Breast Cancer Models. BMC Med. 2012, 10 (1), 1–8. https://doi.org/10.1186/1741-7015-10-80.

(3) Pantziarka, P.; Sukhtame, V.; Meheus, L.; Sukhatme, V. P. V. V.; Bouche, G.; Meheus, L.; Sukhatme, V. P. V. V.; Bouche, G. Repurposing Non-Cancer Drugs in Oncology — How Many Drugs Are out There? bioRxiv 2017. https://doi.org/10.1101/197434.

(4) Tamoxifen. https://www.webmd.com/drugs/2/drug-4497/tamoxifen-oral/details (accessed Aug 29, 2021).

(5) Hofman, J.; Kučera, R.; Cihalova, D.; Klimes, J.; Ceckova, M.; Staud, F. Olomoucine II, but Not Purvalanol A, Is Transported by Breast Cancer Resistance Protein (ABCG2) and P-Glycoprotein (ABCB1). PLoS One 2013. https://doi.org/10.1371/journal.pone.0075520.

(6) Chen, L.; Miao, Y.; Liu, M.; Zeng, Y.; Gao, Z.; Peng, D.; Hu, B.; Li, X.; Zheng, Y.; Xue, Y.; Zuo, Z.; Xie, Y.; Ren, J. Pan-Cancer Analysis Reveals the Functional Importance of Protein Lysine Modification in Cancer Development. Front. Genet. 2018. https://doi.org/10.3389/fgene.2018.00254.

(7) Theraputic Target Database https://db.idrblab.org/ttd/target/t48069 (accessed Jul 27, 2019).

(8) Himmel, L. E.; Lustberg, M. B.; DeVries, A. C.; Poi, M.; Chen, C. S.; Kulp, S. K. Minocycline, a Putative Neuroprotectant, Co-Administered with Doxorubicin-Cyclophosphamide Chemotherapy in a Xenograft Model of Triple-Negative Breast Cancer. Exp. Toxicol. Pathol. 2016. https://doi.org/10.1016/j.etp.2016.08.001.

(9) Can, G.; Ayvaz, S.; Can, H.; Karaboğa, İ.; Demirtaş, S.; Akşit, H.; Yılmaz, B.; Korkmaz, U.; Kurt, M.; Karaca, T. The Efficacy of Tyrosine Kinase Inhibitor Dasatinib on Colonic Mucosal Damage in Murine Model of Colitis. Clin. Res. Hepatol. Gastroenterol. 2016, 40 (4), 504–516. https://doi.org/10.1016/j.clinre.2015.12.006.

(10) Dey, M.; Kuhn, P.; Ribnicky, D.; Premkumar, V.; Reuhl, K.; Raskin, I. Dietary Phenethylisothiocyanate Attenuates Bowel Inflammation in Mice. BMC Chem. Biol. 2010, 10 (1), 1–2. https://doi.org/10.1186/1472-6769-10-4.

(11) Ye, J. H.; Rajendran, V. M. Adenosine: An Immune Modulator of Inflammatory Bowel Diseases. World J. Gastroenterol. 2009, 15 (36), 4491. https://doi.org/10.3748/wjg.15.4491.

(12) Li, T. T.; Zhang, J. F.; Fei, S. J.; Zhu, S. P.; Zhu, J. Z.; Qiao, X.; Liu, Z. B. Glutamate Microinjection into the Hypothalamic Paraventricular Nucleus Attenuates Ulcerative Colitis in Rats. Acta Pharmacol. Sin. 2014, 35 (2), 185–194. https://doi.org/10.1038/aps.2013.140.

(13) Kuralay, F.; Yildiz, C.; Ozutemiz, O.; Islekel, H.; Caliskan, S.; Bingol, B.; Ozkal, S. Effects of Trimetazidine on Acetic Acid-Induced Colitis in Female Swiss Rats. J. Toxicol. Environ. Heal. - Part A 2003, 66 (2), 169–179. https://doi.org/10.1080/15287390306402.

(14) Wan, P.; Liu, X.; Xiong, Y.; Ren, Y.; Chen, J.; Lu, N.; Guo, Y.; Bai, A. Extracellular ATP Mediates Inflammatory Responses in Colitis via P2 × 7 Receptor Signaling. Sci. Rep. 2016, 6 (1), 1–10. https://doi.org/10.1038/SREP19108.

(15) G, C.; M, C.; D, I.; G, B.; I, P.; S, C.; E, E. Dimethyl Fumarate Reduces Inflammatory Responses in Experimental Colitis. J. Crohns. Colitis 2016, 10 (4), 472–483. https://doi.org/10.1093/ECCO-JCC/JJV231.

(16) Drugbank. https://www.drugbank.ca/drugs/DB12695 (accessed Sep 2, 2021).

(17) Sotomayor, E. A.; Teicher, B. A.; Schwartz, G. N.; Holden, S. A.; Menon, K.; Herman, T. S.; Frei, E. Minocycline in Combination with Chemotherapy or Radiation Therapy in Vitro and in Vivo. Cancer Chemother. Pharmacol. 1992, 30, 377–384.

(18) Li, X.; Yang, H.; Sun, H.; Lu, R.; Zhang, C.; Gao, N.; Meng, Q.; Wu, S.; Wang, S.; Aschner, M.; Wu, J.; Tang, B.; Gu, A.; Kay, S. A.; Chen, R. Taurine Ameliorates Particulate Matter-Induced Emphysema by Switching on Mitochondrial NADH Dehydrogenase Genes. Proc. Natl. Acad. Sci. U. S. A. 2017, 114 (45), E9655–E9664. https://doi.org/10.1073/pnas.1712465114.

(19) Canter, P. Herb, Nutrient and Drug Interactions: Clinical Implications and Therapeutic Strategies. Focus Altern. Complement. Ther. 2010. https://doi.org/10.1111/j.2042-7166.2008.tb01840.x.

(20) Lauric Acid Supplement: Uses, Benefits, Side Effects, Dose, Precautions & Warnings https://www.medicinenet.com/lauric_acid/supplements-vitamins.htm (accessed Oct 11, 2021).

(21) Chen, Y.; Gao, S.; Yan, Y.; … J. Q.-A. journal of; 2018, U. Aerosolized Deferoxamine Administration in Mouse Model of Bronchopulmonary Dysplasia Improve Pulmonary Development. ncbi.nlm.nih.gov 2018, 10 (1), 325–332.

(22) Isoprenaline: Uses, Interactions, Mechanism of Action | DrugBank Online https://go.drugbank.com/drugs/DB01064 (accessed Oct 11, 2021).
